# Point-of-Care Testing as an Influenza Surveillance Tool: Methodology and Lessons Learned from Implementation

**DOI:** 10.1155/2013/242970

**Published:** 2013-04-11

**Authors:** Lisa H. Gren, Christina A. Porucznik, Elizabeth A. Joy, Joseph L. Lyon, Catherine J. Staes, Stephen C. Alder

**Affiliations:** ^1^Department of Family and Preventive Medicine, University of Utah, 375 Chipeta Way, Suite A, Salt Lake City, UT 84108, USA; ^2^Department of Biomedical Informatics, University of Utah, 26 S 2000 E RM 5700, Salt Lake City, UT 84112, USA

## Abstract

*Objectives*. Disease surveillance combines data collection and analysis with dissemination of findings to decision makers. The timeliness of these activities affects the ability to implement preventive measures. Influenza surveillance has traditionally been hampered by delays in both data collection and dissemination. *Methods*. We used statistical process control (SPC) to evaluate the daily percentage of outpatient visits with a positive point-of-care (POC) influenza test in the University of Utah Primary Care Research Network. *Results*. Retrospectively, POC testing generated an alert in each of 4 seasons (2004–2008, median 16 days before epidemic onset), suggesting that email notification of clinicians would be 9 days earlier than surveillance alerts posted to the Utah Department of Health website. In the 2008-09 season, the algorithm generated a real-time alert 19 days before epidemic onset. Clinicians in 4 intervention clinics received email notification of the alert within 4 days. Compared with clinicians in 6 control clinics, intervention clinicians were 40% more likely to perform rapid testing (*P* = 0.105) and twice as likely to vaccinate for seasonal influenza (*P* = 0.104) after notification. *Conclusions*. Email notification of SPC-generated alerts provided significantly earlier notification of the epidemic onset than traditional surveillance. Clinician preventive behavior was not significantly different in intervention clinics.

## 1. Introduction

Influenza causes significant morbidity and mortality in the United States (US), with associated costs of 10–77 billion dollars [1–3]. Current US influenza surveillance activities include the reporting of the percentage of outpatient visits due to influenza-like illness (ILI), reports of laboratory-confirmed influenza hospitalizations and pediatric deaths, pneumonia- and influenza-associated mortality, and viral culture for subtyping [4]. The reporting lag associated with these surveillance measures is 1–4 weeks, which limits the implementation of prevention and control activities [5–7]. This has led to investigation of alternate data sources and analytic techniques that provide earlier notification of influenza activity. An article reviewing the timeliness of alternate data sources for influenza surveillance found that over-the-counter pharmaceutical sales, emergency visits, absenteeism, and health advice calls appeared to be more timely than ILI reporting or virological confirmation, by 3–24 days [8]. This lag results from both the timing of the surveillance event relative to the epidemic onset (affecting viral culture results, hospitalizations, and deaths) and the delays inherent in systems that report once weekly (affecting all systems). Another study found that rapid influenza test positivity (the percentage of positive tests among all tests performed) increased 2–5 weeks prior to epidemic notification from ILI surveillance [9].

Outpatient illness, in ambulatory clinics and urgent care settings, is generally the first recognized in the course of an influenza outbreak and is best suited as an early indicator of disease [5, 6, 10]. Currently, outpatient influenza surveillance involves weekly reporting to the Centers of Disease Control (CDC) of the percentage of visits that are due to ILI from approximately 1300 outpatient care sites from all 50 states in the United States (US) [11]. Since the first systematic reports of ILI by outpatient providers for the 1982-83 season [12], little of the methodology has changed and several challenges exist. First, while some outpatient care sites now use electronic records for ILI data collection, many still use paper-based data collection, which can be labor intensive. Second, data transmission to CDC occurs in the week following data collection and is not publicly posted until the end of that week, creating a lag that diminishes the window of opportunity for implementing preventive measures. Third, the ILI case definition is not specific to influenza but can be associated with a variety of respiratory infections, potentially producing a false positive signal.

The University of Utah Primary Care Research Network (UUPCRN) has 10 outpatient clinics and has participated in ILI surveillance with the Utah Department of Health (UDOH) and CDC since 2004. The impetus for our study was the inherent lag in the current ILI reporting system, and a variety of studies that suggested alternative data sources for surveillance could reduce this lag [9, 12–15]. Decreased lag could potentially create a window within which prevention and control activities might be conducted. Using point-of-care (POC) test positivity as an early indicator of influenza activity, we retrospectively analyzed 4 seasons of influenza and created an alerting algorithm based on statistical process control (SPC) charting. In the 5th season, we implemented the algorithm prospectively and evaluated the behavior of clinicians related to influenza identification and prevention in intervention clinics (received email notification of the early alert) compared with control clinics. Finally, we comment on key lessons for implementing an effective and timely clinic-based surveillance and feedback system.

## 2. Methods

### 2.1. Study Population

The UUPCRN is a population-based research network of 10 outpatient clinics and an associated urgent care located in the Salt Lake metropolitan area, where about 2 million of the state's 2.5 million residents live [16, 17]. In fiscal year 2006-07, there were 320,000 UUPCRN outpatient visits, with roughly one quarter of visits occurring among each of 4 age groups: 0–18, 19–35, 36–55, and 56 years and over. Implementation of an electronic medical record (EMR) designed by Epic Systems Corporation (Verona, WI) was begun in 2000. Full operation with the EMR was complete for 6 clinics in 2004-05, 7 in 2005-06, and all 10 for the remaining study years. The number of outpatient visits recorded in the EMR and available for analysis during influenza season (October–May) ranged from 139,303 in 2004-05 to 191,842 in 2007-08.

### 2.2. Influenza Study Period

In the US, influenza surveillance begins in early October and runs through mid-May of the following year, defined by Morbidity and Mortality Weekly Report (MMWR) weeks 40 through 20 [11]. All outpatient visits recorded in an EMR for the UUPCRN patient population were evaluated retrospectively for the seasons 2004-05 through 2007-08. Prospective evaluation of the alerting system and associated clinician behaviors was completed for the 2008-09 season.

### 2.3. Retrospective Analysis

#### 2.3.1. Selection of Alternative Surveillance Measure

The majority of US primary care providers report using influenza tests for ILI patients, most of which are rapid tests [13]. The median sensitivity of influenza rapid tests is 70%–75%, and median specificity is 90%–95% [14]. By comparison, studies of the most predictive syndromic definition (fever plus cough) reported sensitivity of 64%–78% and specificity of 55%–67%, suggesting that rapid tests may have better predictive characteristics than a syndromic case definition [18, 19]. Further, most rapid influenza tests can be conducted at the POC, eliminating the lag that accompanies off-site testing of patient samples. We selected POC testing with a rapid influenza test as our alternative surveillance measure.

Rapid influenza tests were introduced into the UUPCRN clinics in December 2003 and were used in conjunction with viral culture for the remainder of the 2003-04 influenza season. In all subsequent seasons, UUPCRN clinics used POC rapid tests almost exclusively. Beginning with the 2003-04 influenza season, UDOH began accepting rapid tests, in addition to viral culture, as laboratory confirmation for influenza surveillance [20].

#### 2.3.2. Clinical Measures

The UUPCRN clinics contributed influenza surveillance data to UDOH, as part of the Sentinel Providers Network. The CDC case definition for ILI is

- fever of 100°F (37.8°C) or higher,
- with cough and/or sore throat,
- in the absence of another known cause for these symptoms [11].

The implemented UUPCRN case definition for ILI required a measured temperature of 100°F (37.8°C) or higher and a chief complaint code of cough or sore throat or both as the reason for the visit. For comparison of UUPCRN daily ILI surveillance with traditional weekly ILI surveillance, UDOH provided weekly ILI data collected for the CDC's Sentinel Providers Network for the seasons 2004-05 through 2007-08.

POC testing was completed on the same day that patients were swabbed. Test results were entered into the patient's EMR by the laboratory technician on the same day. An automated report extracted information from the EMR and provided daily counts of the number of outpatient visits to UUPCRN clinics, the number of patients who met the ILI case definition, and the number who had a positive rapid influenza test. Using the number of daily patient visits as the denominator, the proportion of patients with ILI and the proportion of patients with a positive rapid influenza test were calculated for each day during the influenza season for the seasons 2004-05 through 2007-08.

#### 2.3.3. Statistical Methods

SPC methodology was applied to the daily proportion of ILI visits and of positive rapid influenza tests to build seasonal charts using Statit Custom QC (Statware, Inc.). Process control charts generated a mean proportion (or center line) for each clinical indicator and confidence limits (or control limits) that defined the bounds for normal variation. We selected an upper control limit of 3 standard deviations (or 3 sigma) above the center line as the threshold for generating an alert of increased influenza activity. The upper control limit was calculated separately for weekends and weekdays, due to the lower number of patient visits on weekends when only the urgent care facility was open. Smoothed graphs were generated using a 7-day moving average.

Timeliness was measured as the number of days between an early alert and the beginning of the influenza epidemic curve. Using the Western Electric Rules for identifying an action signal, the date on which 4 of 5 consecutive points exceeded the 1 sigma limit was defined as the epidemic onset [21]. Early alerts were generated for both retrospective and prospective viewpoints of the season. Full-season charting (retrospective) used an entire season's data to create the SPC chart, and a full-season early alert was defined as the first signal exceeding the upper control limit prior to the epidemic onset.

Alternatively, we built the SPC chart one day at a time, to simulate real-time surveillance (prospective). Under this scenario, there were multiple single-day alerts. Because these were potentially false alarms, we defined a real-time surveillance alert as a point that exceeded the upper control limit followed by a point that did not return to the center line. The date of this second point defined the real-time surveillance early alert.

Timeliness of SPC in the UUPCRN system was compared with other surveillance systems available to Utah clinicians. These included influenza surveillance data posted to both the CDC and UDOH websites [11, 22]. The data for each of these are reported once weekly for the MMWR weeks, defined as Sunday through Saturday [23]. The Utah Sentinel Providers send their data to UDOH, where the data are posted on the Wednesday following the end of the data collection week, resulting in a 4-day lag after the close of the data collection week. The UDOH forwards these reports to CDC, where weekly data for all US Sentinel Providers are posted to CDC's website on the Friday following the end of the data collection week, resulting in a 6-day lag after the close of the data collection week. The web posting dates were defined as the notification date for the UDOH and CDC systems. The UUPCRN data were available the day following the clinic visit. Assuming a 24-hour time frame to analyze the data and send an email to clinicians, the potential UUPCRN notification date was defined as 2 days after generation of the real-time surveillance alert. This date was compared with the date UDOH and CDC posted surveillance results on their websites.

### 2.4. Prospective Analysis

#### 2.4.1. Identification of Early Alert

For the 2008-09 season, we implemented daily prospective charting of the proportion of patients with a positive rapid test. Alerts were defined using the same methods described in the retrospective section (aforementioned). The early alert and the epidemic onset dates created the following time periods: prealert, early alert, and epidemic.

#### 2.4.2. Early Alert Notification to Providers

The UUPCRN clinics were categorized as academic clinics where residency training occurs (*n* = 2), and community clinics were stratified by annual patient volume (2 high volume, 4 intermediate volume, and 2 small volume). Within each of these 4 strata, one clinic was randomized to receive early notification by email of the influenza alert and designated as intervention clinics. The remaining 6 control clinics did not receive the email, representing 56% of total patient visits. This research was approved by the University of Utah IRB.

Our plan was for the UUPCRN medical director to send an email to clinicians in intervention clinics when the early alert was noted in the SPC chart. The email would identify the date of the early alert and, based on historical data, estimate the date for the epidemic onset. The email would also include additional messages based on information from the Centers for Disease Control and Prevention (CDC) website [24, 25]:

- recommending vaccination because protective antibodies develop within 2 weeks after vaccination;
- providing guidance on antiviral therapy and prophylaxis.

#### 2.4.3. Statistical Analysis

The reason for visit, chief complaint(s), and vital signs were abstracted from the EMR to classify patients visits as ILI or non-ILI. Orders for rapid influenza testing, influenza immunization, and antiviral prescriptions were abstracted from the EMR to assess the impact of the intervention on clinician behavior. The proportion of influenza-associated visits with a particular clinician behavior was calculated. To quantify the association between clinics receiving early notification and subsequent clinician behavior, logistic regression was used. An interaction term was created for intervention status and the time period prior to the epidemic onset (prealert and early alert). The 95% confidence intervals (CIs) and *P* values for the interaction odds ratios (ORs) were used to determine whether a statistically significant difference for clinician behavior was evident for intervention clinicians during the early alert period. Analyses were conducted using Stata, version 8.2 (StataCorp, College Station, TX).

## 3. Results

### 3.1. Retrospective Analysis: Full-Season SPC Charting

**Figure 1:**
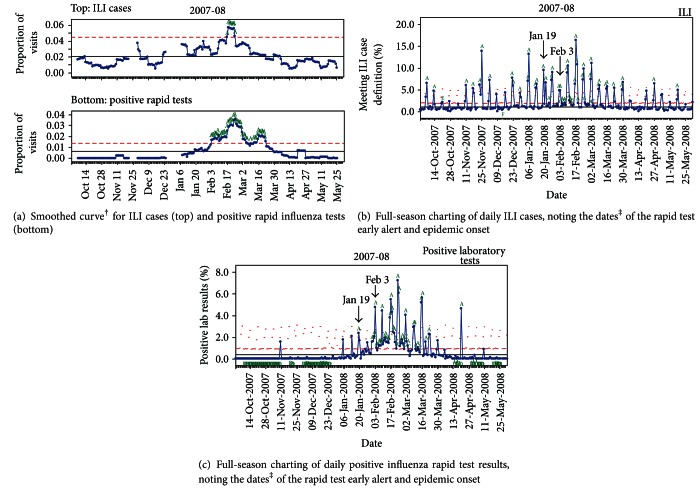
Full-season charting of ILI cases and positive rapid influenza tests using statistical process control* of daily proportions in the UUPCRN population for the 2007-08 influenza season. *Solid horizontal lines define the center line, and dashed lines define the upper control limit. Letters on the chart denote particular events: “A” is 1 point above 3 sigma; “C” is 9 points in a row above the center line; “D” is 9 points in a row below the center line; “E” is 6 points in a row that are increasing (or decreasing); “F” is 14 points in a row alternating up and down. ^†^7-day smoothed graphs have 2 gaps of 7 days each, corresponding to single day clinic closures for Thanksgiving (Nov 22) and Christmas (Dec 25). ^‡^Early alert (Jan 19) was the date of the modeled real-time surveillance alert for positive rapid tests; epidemic onset (Feb 3) was the date when 4 of 5 consecutive days exceeded 1 sigma for positive rapid tests.

The SPC charts for full-season charting of UUPCRN data are provided for the 2007-08 season in Figure 1. Similar graphs were obtained for seasons 2004-05 through 2006-07 (data not shown).

The smoothed curve generated with a 7-day moving average is shown for ILI (upper graph) and rapid test positivity (lower graph) in Figure 1(a). The horizontal lines define the center line (solid) and upper control limit (dashed), with data points exceeding the upper control limit labeled “A.” Over the 4 seasons, the epidemic onset occurred once in December and January and twice in February (data not shown for the remaining 3 seasons). Although the epidemic onset occurred in different months, several trends emerged from the 7-day moving average graphs. First, rapid test and ILI peaks coincided in time, although rapid test peaks were of longer duration. Second, the rapid test alert started earlier than the ILI alert in all 4 seasons (range: 3–35 days). Third, the rapid test data defined more than 1 peak in all 4 seasons while ILI defined a single peak each season. In 2 seasons, the magnitude of these additional peaks was similar to the main peak. In 1 season, the additional peaks actually preceded the main peak.

The SPC charts for full-season charting of daily ILI and positive rapid influenza tests in UUPCRN are provided for the 2007-08 season in Figures 1(b) and 1(c), respectively. Using the daily positive rapid influenza tests data (Figure 1(c)), the early alert occurred on January 19th. The epidemic onset occurred on February 3rd, as defined by the Western Electric Rules [21]. These dates are identified with arrows in Figures 1(b) and 1(c). By contrast, there was no clear epidemic peak evident on the ILI graph and the percentage of visits due to ILI-generated signals through the entire season, with 10 false alerts generated prior to January 19th. Over the 4 seasons, the number of false alerts on the ILI graph ranged from 5 to 10 (data not shown).

### 3.2. Retrospective Analysis: Modeling Real-Time Charting

**Figure 2:**
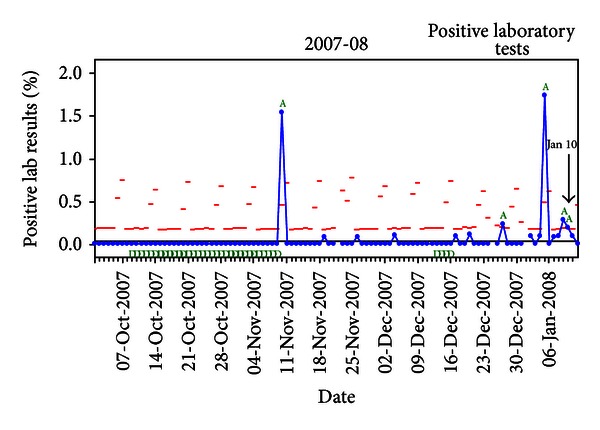
Statistical process control chart* of modeled real-time surveillance of positive influenza rapid tests demonstrating an early alert in the UUPCRN population for the 2007-08 season. *Solid horizontal line defines the center line. Dashes above each data point represent the upper control limit calculated for that day. Points exceeding the upper control limit are noted by “A.” The modeled real-time surveillance alert (Jan 10) was defined as the date for the data point following an exceedance of the upper control limit (A) that does not subsequently return to the center line.

The real-time surveillance graph of positive laboratory tests for the 2007-08 season is shown in Figure 2.

The modeled real-time surveillance alert occurred on January 10th (noted by an arrow), 9 days earlier than the alert generated in the full-season chart. Alerts for modeled real-time surveillance occurred earlier than full-season charting in all 4 seasons. A comparison of the alert dates for full-season charting and real-time surveillance for each season is provided in Table 1.

The mean number of days that the early alert preceded the epidemic onset was 7.0 days for full-season charting and 15.5 days for modeled real-time surveillance (*P* = 0.039 for a one-sided *t*-test comparing these means).

**Figure 3:**
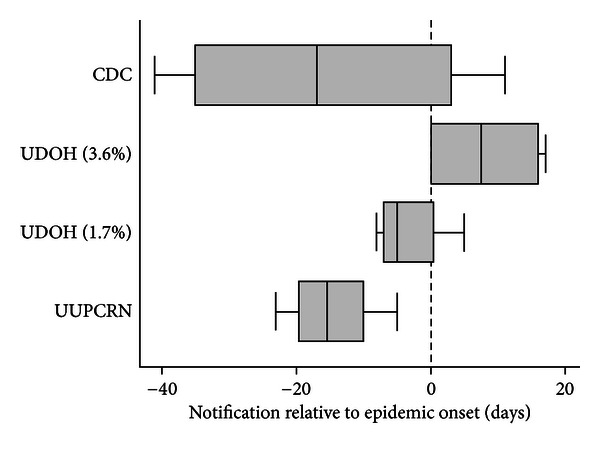
Distribution of notification dates* of increased influenza activity relative to the UUPCRN epidemic onset by notification source^†^ for seasons 2004-05 through 2007-08. *Notification dates were based on the date that Sentinel Physician ILI reports were posted to the CDC and UDOH websites and the theoretical notification by email to UUPCRN clinicians 2 days following the early alert signal. ^†^Notification source: CDC—Centers for Disease Control and Prevention. The national ILI threshold ranged from 2.1% to 2.5% during the study years. UDOH—Utah Department of Health. The historical Utah-specific ILI threshold was 3.6% during the study years and is shown as the first UDOH group in the graph. The threshold was recalculated in 2008-09 to 1.7% and is shown as the second UDOH group. The 1.7% threshold demonstrates the shift in notification date if this threshold had been used in the study years. UUPCRN—University of Utah Primary Care Research Network. The notification date is based on a theoretical email notification sent to clinicians 2 days after the modeled real-time surveillance alert.

Utah clinicians can access the UDOH or CDC websites for updates on ILI, with notification dates defined for this comparison as the day on which the new weekly surveillance summary was posted. The notification dates for CDC and UDOH were compared with a theoretical notification date to UUPCRN clinicians 2 days following the real-time surveillance alert. Box plots for website posting in all 4 seasons for CDC, UDOH, and the estimated UUPCRN notification are provided in Figure 3.

Two alerting thresholds for UDOH were used for comparison, corresponding to the Utah-specific ILI baseline calculated during the years of the study (3.6% of visits due to ILI) and the revised Utah-specific baseline (1.7% of visits due to ILI) that was used for the 2008-09 and later seasons. The latter baseline was closer to the regional baseline of 1.5%–1.6% and is expected to be more accurate. The UUPCRN theoretical notification date always occurred prior to the epidemic onset (median: −14.0 days). While the median notification date occurred prior to the epidemic onset for both the CDC and the revised UDOH (1.7%) systems (−18.0 and −5.0 days, resp.), each had a notification date after the UUPCRN epidemic onset during 1 season. The UDOH (3.6%) notification occurred after the UUPCRN epidemic onset in 2 seasons (median: 16 days) and never exceeded the ILI threshold in the other 2 seasons.

### 3.3. Prospective Analysis: Implementation

**Figure 4:**
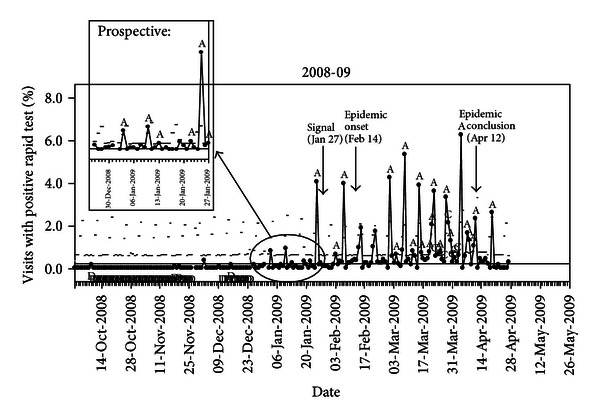
Statistical process control chart* of the percent of visits with a positive rapid influenza test in UUPCRN clinics during the 2008-09 season. *Circled portion of the graph represents part (Dec 25–Jan 27) of the prealert period and includes the early alert signal. The signal occurred on Jan 26 (data point that did not return to baseline following a 3 sigma exceedance), with the data download on Jan 27, making the latter date the signal available to the analyst. Symbols used in the graph are A—designates a data point exceeded the upper control limit (3 sigma), C—designates the 9th data point in a row above the center line, and D—designates the 9th data point in a row below the center line.

The SPC graph for the 2008-09 influenza season is provided in Figure 4, with detail for the month preceding the alert shown in the inset.

The SPC alert of increased influenza activity in the clinics occurred on January 26th. The time periods defined by the SPC chart were prealert (Oct 1, 2008–Jan 26, 2009), early alert (Jan 27–Feb 13, 2009), and epidemic period (Feb 14–Apr 12, 2009). For 2008-09, the early alert occurred 19 days prior to the epidemic onset. Nationally and in Utah, the 2008-09 season was mild, prior to the circulation of 2009 H1N1 in the late spring. This is reflected in the SPC chart where the epidemic onset is followed by sporadic activity after the alert rather than the usual epidemic curve (as seen in Figure 1(c)).

The early signal was identified on January 27th, and an email was prepared on January 28th, which was consistent with our plan to notify clinicians within 2 days. However, due to delays in administrative processing, the email was not delivered to intervention clinicians until January 30th. Therefore, actual time from alert to notification was 4 days. Even with the email delay, intervention clinicians were notified 15 days prior to the epidemic onset, representing a time during which they might have implemented prevention and control measures for their patients at risk for influenza infection.

### 3.4. Prospective Analysis: Clinician Behaviors

**Figure 5:**
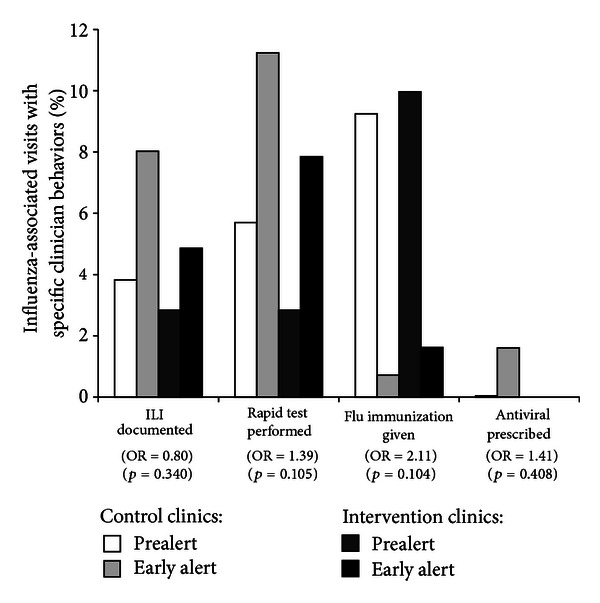
The percentage of influenza-associated outpatient visits with particular clinician behaviors in 2008-09, by intervention status for the prealert and early alert time periods*. *The odds ratio (OR) and associated *P* value are reported for the interaction term (intervention status and time period) in the logistic regression model.

Among patients with an influenza-associated visit during the prealert and early alert periods, 4.1% (range: 2.8%–8.0%) had sufficient information documented in the EMR to code them as ILI cases for traditional surveillance; 5.6% (range: 2.8%–11.2%) had a rapid test performed; 7.9% (range: 0.7%–10.0%) received an influenza immunization; 0.3% (range: 0.04%–1.6%) received a prescription for an antiviral medication (see Figure 5).

As expected, influenza immunizations were higher in the prealert period (October–January), corresponding with the timeframe when seasonal immunization for influenza is emphasized. Other behaviors were higher in the early alert than in the prealert period, which was expected since they were responses to higher levels of respiratory illness including influenza.

We used logistic regression to model the combined impact of notification status and time period for each clinician behavior. The interaction term compared clinician behavior in intervention clinics during the early alert period relative to the prealert period, as well as to clinicians in control clinics during both time periods. While not statistically significant at alpha = 0.05, the ORs for clinician behaviors (Figure 5) suggest that intervention clinicians were 40% more likely to perform rapid testing (OR = 1.39, 95% CI = 0.93–2.08) and twice as likely to immunize against influenza (OR = 2.11, 95% CI = 0.86–5.18) during the early alert period than their counterparts.

## 4. Discussion

### 4.1. Statistical Process Control as an Early Alert Tool: Methodology and Implementation

We used SPC charting to monitor the daily percentage of patient visits with a positive rapid influenza test identified from the EMR of a population-based network of outpatient clinics. Building the charts to simulate real-time surveillance, an early alert was identified for each of 4 seasons, occurring a median of 16 days prior to the epidemic onset. We estimated that notification of this early alert could be sent by email to UUPCRN clinicians within 2 days of the alert. By comparison, our hypothesized notification date was 9 days earlier than the UDOH website posting date. The median notification date for the CDC website posting was similar to our early alert but had wider variability, including one season when CDC notification occurred after the UUPCRN epidemic onset. In actual implementation during the 2008-09 season, the early alert occurred 19 days before the epidemic onset, but notification took 4 days rather than the hypothesized 2 days. There were increases in clinician performance of rapid testing and antiviral prescribing practicing at intervention clinics during the early alert period, but neither was statistically significant.

Other studies evaluating the timeliness of early signals in outpatient populations have been conducted and report a lead time of 7–24 days. Three studies have used alternative modeling techniques with ILI data and demonstrated earlier signals relative to traditional ILI surveillance [26–28]. A fourth study reported that the proportion of clinical visits with a positive rapid influenza test result began rising 2–5 weeks earlier than the proportion of ILI [9]. Our finding of a 16-day lead time for the real-time surveillance is similar to these other studies in outpatient settings. Further, our analytic method is relatively simple to implement and interpret visually, making it attractive to use in clinical settings that do not have access to the statistical expertise required to perform sophisticated modeling, as required by some of these studies.

The use of rapid influenza test results stored in an EMR has several potential advantages over the identification of ILI cases for surveillance. First, rapid influenza tests are reported to have comparable sensitivity and higher specificity than a syndromic definition of fever and cough [14, 18, 19]. Consequently, use of rapid influenza tests for surveillance could result in fewer false positive signals. Second, the influenza test results are stored in the EMR as part of usual patient care while identification of ILI cases requires the use of a non-patient-related data collection form. Thus, data captured from the EMR is likely to be less labor intensive and more complete. Health departments in Hawaii and Indiana that used automated electronic laboratory reporting have documented 2.3- and 4.4-fold increases in reporting, respectively [29, 30]. These same studies also demonstrated that reports were received 3.8 and 7.9 days earlier compared to paper-based methods. Third, the EMR data are available daily rather than weekly, enhancing the timeliness of analysis and notification and providing a window within which preventive activities could be initiated.

Several potential limitations may affect the interpretation of our findings. It is possible that the UUPCRN study population is not representative of the general population and that an early signal generated in our data would not reflect the timing of influenza activity in the broader population. We compared the UUPCRN rapid influenza test results with ILI reporting for both UUPCRN and UDOH [15], as well as with respiratory culture findings from the GermWatch laboratory surveillance system in Utah [31]. The clinical and laboratory influenza indicators increased and decreased over the same time period for the seasons 2004-05 through 2007-08, suggesting that the UUPCRN data are representative of the population of the Salt Lake metropolitan area [15]. Further, in 3 of the seasons the GermWatch data demonstrated that the respiratory syncytial virus (RSV) epidemic started 3–7 weeks before the influenza epidemic, but our early alert signal coincided with the GermWatch influenza epidemic and did not give a false signal associated with the earlier RSV epidemic.

It is also possible that the use of influenza testing among UUPCRN clinicians differs from their peers in Utah. If UUPCRN clinicians were more likely to use rapid testing, especially early in the season when the risk of false positive results is higher, our early signal based on test results might reflect false positives. Differential use of influenza testing seems unlikely because UDOH reported that 86% of 6,340 laboratory-confirmed influenza cases for the 2003-04 season were diagnosed by rapid test, suggesting that use of rapid tests is widespread [32]. Investigation of the impact of our rapid test analyses on practices outside UUPCRN was beyond the scope of this study.

Because of their relative ease of acquisition, throat swabs are the most common samples available for rapid testing but generally have lower sensitivity than other clinical samples [33]. It is possible that rapid tests in UUPCRN were conducted predominantly using throat swab samples, which could further compound the problem of a high rate of false negative tests early in the season when the prevalence of influenza is low. We minimized the possibility of a false positive early signal by defining our real-time surveillance alert as a peak that contained a point exceeding the upper control limit followed by a point that did not return to the center line rather than using a single point for the alert. Based on this conservative definition, the timeliness of our early alert was similar to that seen in other studies [26–28]. However, further study using a confirmatory test such as viral culture or polymerase chain reaction would be required to determine whether these earlier peaks in our population are false positives or represent the earliest circulation of influenza in the population.

### 4.2. Lessons Learned

Our study demonstrated that POC influenza testing generated an early alert signal, averaging 16 days prior to the epidemic onset of influenza in the community. When this surveillance system was implemented prospectively, intervention clinicians received notification 15 days prior to the epidemic onset. In the outpatient setting, dissemination of relevant influenza surveillance information has the potential to aid clinicians in case management [14, 34]. We learned several lessons from our year of implementation that relate to the surveillance metrics, clinician notification, and the value of POC testing as a surveillance measure.

In the mild 2008-09 season, the epidemic onset was difficult to recognize visually from the prospective graphic; however, the surveillance metric (based on the Western Electric rules [21]) we used provided an identifiable early alert, as well as start to the epidemic period. By contrast, ILI never exceeded the Utah-specific threshold defining an outbreak [35]. In hindsight, lack of a typical epidemic curve seen in the SPC graph of the 2008-09 season may have been an early indication of the unusual season. In the future, curves deviating from the expected shape or timeframe should prompt investigation to determine potential reason(s) for the deviation. The goal of our study was to return surveillance information to clinicians, but we did not create an avenue for clinicians to return any clinical observations that were not part of the automatically extracted data. Given the atypical 2008-09 season, clinicians may have had insights that would have been important in an investigation of the unusual shape of the epidemic curve. Developing a forum for this dialog is an area for further research.

Studies have reported that clinician perception of the care they provide, as well as actual diagnosis and use of resources, is improved when surveillance data are available to them [18, 19, 28, 34, 36–38]. However, we did not find significant differences between intervention and control clinics with regard to the clinician behaviors we measured. It is possible that our nonsignificant results are a result of one or a combination of factors associated with our messaging practices. First, our email message was sent through an intranet limiting the message to a text summary. A study of Utah clinicians documented a preference for time series graphs over textual summaries to represent trends for respiratory pathogens [39]. Another limitation was that the message was only sent once. Clinician preference for frequency of such notifications has varied from weekly to quarterly when an outbreak occurs and likely reflects the fact that different circumstances require different notification frequencies [40–42]. Finally, we did not measure receipt of the email, whether it was opened, understood, agreed with, diffused to other clinical staff, or whether and when the prevention recommendations were implemented. Additional evaluation of the format and frequency of data delivery, as well as clinician knowledge, opinions, and behaviors related to receipt of surveillance messages, appears warranted to determine the optimal circumstances for providing relevant information that improves clinical decision-making.

The earlier notification from rapid test positivity, coupled with its ability to identify the epidemic period even in seasons when ILI never exceeded the threshold, suggests that it is a more accurate surveillance measure than ILI. The ILI clinical case definition was first implemented for the 1982-83 season, prior to the availability of point-of-care influenza testing, and no substantive changes have been made in outpatient surveillance in the interim [12]. One of the main concerns voiced about using rapid influenza tests is the low sensitivity (about 70%), resulting in poor predictive value when the prevalence is low, that is, early in the season [33, 37]. However, at a population level, rapid test positivity in UUPCRN clinics was a consistently better indicator of the onset of the epidemic period than ILI. Our findings suggest two changes to improve surveillance for future influenza seasons: (1) a shift in the use of rapid influenza tests from diagnosis to surveillance and (2) a shift in influenza surveillance from ILI to rapid test positivity. Results from our study were based on rapid test positivity as the test was used in clinical practice; further research is needed to determine the conditions (e.g., sampling strategies for clinicians and patient identification) under which this test would be used as a surveillance tool.

## Figures and Tables

**Table 1:** Comparison of early alert dates identified by statistical process control charts for full-season charting and modeled real-time charting of positive rapid influenza tests in the UUPCRN population for 4 seasons.

Influenza season	Date epidemic started	Full-season charting		Modeled real-time charting	
		Early alert* date relative to epidemic onset (days)	No. visits to trigger early alert* (% of visits)	Early alert^†^ date relative to epidemic onset (days)	No. visits to trigger early alert^†^ (% of visits)
2004-05	Jan 13	−13	2 (2.7%)	−17	2 (0.3%)
2005-06	Dec 13	−2	3 (3.6%)	−6	2 (0.2%)
2006-07	Feb 2	+2	2 (2.4%)	−15	4 (0.4%)
2007-08	Feb 3	−15	3 (2.4%)	−24	2 (0.2%)

Mean^‡^ (sd)		−7.0 (8.3)		−15.5 (7.4)	
Median		−7.5		−16.0	

*Early alert occurred on the date that rapid test positivity exceeded 3 sigma.

^†^Early alert occurred on the date that rapid test positivity exceeded 3 sigma and did not return to the center line on the following day.

^‡^One-sided *t*-test of means was significant (*P* = 0.039).

## References

[1] Thompson WW, Shay DK, Weintraub E (2003). Mortality associated with influenza and respiratory syncytial virus in the United States. *Journal of the American Medical Association*.

[2] Thompson WW, Shay DK, Weintraub E (2004). Influenza-associated hospitalizations in the United States. *Journal of the American Medical Association*.

[3] Molinari NAM, Ortega-Sanchez IR, Messonnier ML (2007). The annual impact of seasonal influenza in the US: measuring disease burden and costs. *Vaccine*.

[4] Centers for Disease Control and Prevention Overview of influenza surveillance in the United States. http://www.cdc.gov/flu/weekly/pdf/overview.pdf.

[5] Matlof H, Murray RA, Kamei I, Heidbreder GA (1971). Influenza in Los Angeles County, 1968-69. *HSMHA Health Reports*.

[6] Quenel P, Dab W, Hannoun C, Cohen JM (1994). Sensitivity, specificity and predictive values of health service based indicators for the surveillance of influenza A epidemics. *International Journal of Epidemiology*.

[7] Miller B, Kassenborg H, Dunsmuir W (2004). Syndromic surveillance for influenzalike illness in an ambulatory care network. *Emerging Infectious Diseases*.

[8] Dailey L, Watkins RE, Plant AJ (2007). Timeliness of data sources used for influenza surveillance. *Journal of the American Medical Informatics Association*.

[9] Baumbach J, Mueller M, Smelser C, Albanese B, Sewell CM (2009). Enhancement of influenza surveillance with aggregate rapid influenza test results: New Mexico, 2003–2007. *American Journal of Public Health*.

[10] Lazarus R, Kleinman K, Dashevsky I (2002). Use of automated ambulatory-care encounter records for detection of acute illness clusters, including potential bioterrorism events. *Emerging Infectious Diseases*.

[11] Centers for Disease Control and Prevention http://www.cdc.gov/flu/weekly/fluactivity.htm.

[12] Centers for Disease Control and Prevention (1983). Current trends influenza surveillance summary—United States, 1982-1983 season. *Morbidity and Mortality Weekly Report*.

[13] Centers for Disease Control and Prevention (2008). Influenza-testing and antiviral-agent prescribing practices-Connecticut, Minnesota, New Mexico, and New York, 2006-07 influenza season. *Morbidity and Mortality Weekly Report*.

[14] World Health Organization WHO recommendations on the use of rapid testing for influenza diagnosis. http://www.who.int/csr/disease/avian_influenza/guidelines/RapidTestInfluenza_web.pdf.

[15] Gren LH (2010). *Outpatient influenza surveillance: bridging the gap between between clinical medicine and public health [dissertation]*.

[16] US Census http://factfinder.census.gov.

[17] US Census State & County QuickFacts. http://quickfacs.census.gov.

[18] Monto AS, Gravenstein S, Elliott M, Colopy M, Schweinle J (2000). Clinical signs and symptoms predicting influenza infection. *Archives of Internal Medicine*.

[19] Boivin G, Hardy I, Tellier G, Maziade J (2000). Predicting influenza infections during epidemics with use of a clinical case definition. *Clinical Infectious Diseases*.

[20] Utah Department of Health Influenza summary report 2005-06 season. http://health.utah.gov/epi/disease/flu/ClinicianPublicHealth/PH_DiseaseStatus/u2005/influseasonSum.pdf.

[21] Montgomery DC (2005). *Introduction to Statistical Quality Control*.

[22] Utah Department of Health Utah public health influenza surveillance program. http://www.health.utah.gov/epi/diseases/flu/SurvInfo.htm.

[23] Centers for Disease Control and Prevention MMWR week fact sheet. http://www.cdc.gov/ncphi/disss/nndss/phs/mmwrweek/MMWR_Week_Fact_Sheet.doc.

[24] Centers for Disease Control and Prevention Seasonal flu vaccine. http://www.cdc.gov/flu/about/qa/fluvaccine.htm.

[25] Centers for Disease Control and Prevention Interim recommendations for the use of influenza antiviral medications in the setting of oseltamivir resistance among circulating influenza A (H1N1) viruses, 2008-09 influenza season. http://www.cdc.gov/flu/professionals/antivirals/.

[26] Miller B, Kassenborg H, Dunsmuir W (2004). Syndromic surveillance for influenza like illness in an ambulatory care network. *Emerging Infectious Diseases*.

[27] Ritzwoller DP, Kleinman K, Palen T (2005). Comparison of syndromic surveillance and a sentinel provider system in detecting an influenza outbreak–Denver, Colorado, 2003. *Morbidity and Mortality Weekly Report*.

[28] Nagykaldi Z, Mold JW, Bradley KK, Bos JE (2006). Bridging the gap between public and private healthcare: influenza-like illness surveillance in a practice-based research network. *Journal of Public Health Management and Practice*.

[29] Effler P, Ching-Lee M, Bogard A, Ieong MC, Nekomoto T, Jernigan D (1999). Statewide system of electronic notifiable disease reporting from clinical laboratories: comparing automated reporting with conventional methods. *Journal of the American Medical Association*.

[30] Overhage JM, Grannis S, McDonald CJ (2008). A comparison of the completeness and timeliness of automated electronic laboratory reporting and spontaneous reporting of notifiable conditions. *American Journal of Public Health*.

[31] Intermountain Germ Watch Respiratory Virus Surveillance. https://intermountain.net/portal/site/mdvsi.

[32] Utah Department of Health http://health.utah.gov/epi/newsletter/04may/May_2004_newsletter.pdf.

[33] Gavin PJ, Thomson RB (2004). Review of rapid diagnostic tests for influenza. *Clinical and Applied Immunology Reviews*.

[34] Bonner AB, Monroe KW, Talley LI, Klasner AE, Kimberlin DW (2003). Impact of the rapid diagnosis of influenza on physician decision-making and patient management in the pediatric emergency department: results of a randomized, prospective, controlled trial. *Pediatrics*.

[35] Utah Department of Health http://health.utah.gov/epi/diseases/influenza/surveillance/2008-09_Season/PH&C_042909_wk16_flu_posting.pdf.

[36] Gesteland PH, Allison MA, Staes CJ Clinician use and acceptance of population-based data about respiratory pathogens: implications for enhancing population-based clinical practice.

[37] Grijalva CG, Poehling KA, Edwards KM (2007). Accuracy and interpretation of rapid influenza tests in children. *Pediatrics*.

[38] Falsey AR, Murata Y, Walsh EE (2007). Impact of rapid diagnosis on management of adults hospitalized with influenza. *Archives of Internal Medicine*.

[39] Gesteland PH, Samore MH, Pavia AT Informing the front line about common respiratory viral epidemics.

[40] Krause G, Ropers G, Stark K (2005). Notifiable disease surveillance and practicing physicians. *Emerging Infectious Diseases*.

[41] Janssen AP, Tardif RR, Landry SR, Warner JE (2006). “Why tell me now?” The public and healthcare providers weigh in on pandemic influenza messages. *Journal of Public Health Management and Practice*.

[42] Gren LH, Alder SC, Joy EA (2008). *Clinicians’ Preferred Method of Communication in a Practice-Based Research Network*.

